# Prior infection with Type A *Francisella tularensis* antagonizes the pulmonary transcriptional response to an aerosolized Toll-like receptor 4 agonist

**DOI:** 10.1186/s12864-015-2022-2

**Published:** 2015-10-28

**Authors:** Kathie-Anne Walters, Rachael Olsufka, Rolf E. Kuestner, Xiagang Wu, Kai Wang, Shawn J. Skerrett, Adrian Ozinsky

**Affiliations:** Institute for Systems Biology, 401 Terry Ave. N, Seattle, WA 98109 USA; Division of Pulmonary and Critical Care Medicine, University of Washington, Seattle, WA 98104 USA

## Abstract

**Background:**

*Francisella* infection attenuates immune cell infiltration and expression of selected pro-inflammatory cytokines in response to endogenous LPS, suggesting the bacteria is actively antagonizing at least some part of the response to Toll-like receptor 4 (TLR4) engagement. The ability of different *Francisella* strains to inhibit the ability of *E. coli* LPS to induce a pulmonary inflammatory response, as measured by gene expression profiling, was examined to define the scope of modulation and identify of inflammatory genes/pathways that are specifically antagonized by a virulent *F. tularensis* infection.

**Results:**

Prior aerosol exposure to *F. tularensis* subsp. *tularensis*, but not the live attenuated strain (LVS) of *F. tularensis* subsp. *holarctica* or *F. novicida*, significantly antagonized the transcriptional response in the lungs of infected mice exposed to aerosolized *E. coli* LPS. The response to *E. coli* LPS was not completely inhibited, suggesting that the bacteria is targeting further downstream of the TLR4 molecule. Analysis of the promotors of LPS-responsive genes that were perturbed by Type A *Francisella* infection identified candidate transcription factors that were potentially modulated by the bacteria, including multiple members of the forkhead transcription factor family (FoxA1, Foxa2, FoxD1, Foxd3, Foxf2, FoxI1, Fox03, Foxq1), IRF1, CEBPA, and Mef2. The annotated functional roles of the affected genes suggested that virulent *Francisella* infection suppressed cellular processes including mRNA processing, antiviral responses, intracellular trafficking, and regulation of the actin cytoskeleton. Surprisingly, despite the broad overall suppression of LPS-induced genes by virulent *Francisella*, and contrary to what was anticipated from prior studies, Type A *Francisella* did not inhibit the expression of the majority of LPS-induced cytokines, nor the expression of many classic annotated inflammatory genes.

**Conclusions:**

Collectively, this analysis demonstrates clear differences in the ability of different *Francisella* strains to modulate TLR4 signaling and identifies genes/pathways that are specifically targeted by virulent Type A *Francisella*.

**Electronic supplementary material:**

The online version of this article (doi:10.1186/s12864-015-2022-2) contains supplementary material, which is available to authorized users.

## Background

*Francisella tularensis* is a Gram-negative facultative intracellular bacterium capable of causing severe disease (tularemia) in humans. Type A (*F. tularensis* subsp *tularensis* strains are highly virulent and associated with a severe clinical course, particularly pneumonic tularemia, in North America. Type B (*F. tularensis* subsp *holarctica*) strains cause acute but mild self-limiting infections primarily in Europe. *F. tularensis* subsp *mediastatica* appears to be similar in virulence to type B strains, but is limited to Central Asia. *F. novicida*, sometimes considered another subspecies of *F. tularensis*, is highly virulent in mice but a rare cause of human disease. The Centers for Disease Control has classified Type A. *F. tularensis* as a Tier 1 select agent based on its high infectivity and lethality, multiple routes of infection and potential use in bioterrorism. The bacterium is considered particularly dangerous because of its potential for aerosol transmission, low infectious dose (as few as 10 organisms), and severe morbidity/mortality (up to 30 % mortality rate if untreated) [1, 2]. That combined with the lack of an approved preventative vaccine against pneumonic tularemia, as well as concerns about antibiotic-resistant isolates, has led to renewed interest in this pathogen.

The innate immune response represents the first-line of defense against bacterial infection and plays a key role in the initial pathogen detection and subsequent activation of adaptive immunity. Not surprisingly, bacteria have evolved mechanisms to evade and perturb host defense responses to facilitate their own replication and this often correlates with the level of a pathogen’s virulence. The fundamental molecular processes involved in the extreme virulence of Type A *F. tularensis* are not well understood but studies have demonstrated that it is associated with an absence of a classic inflammatory response. For example, *F. tularensis* fails to stimulate production of pro-inflammatory mediators, including TNF-α and IL12B (IL-12p40,) or activate dendritic cells (DC) in the airways and lungs of aerosol-exposed mice [3]. *F. tularensis* infection of mononuclear phagocytic cells also fails to stimulate IFN-γ [4] or other cytokines [5–7]. More recent studies have shown that inflammasome activation is suppressed during early *Francisella* infection via targeting of TLR2-dependent signaling by the bacterial gene FTL_0235 encoded protein [8]. Nuclear localization of the p65 subunit of NF-kβ was also found to be partially inhibited by FTT031c, partially suppressing pro-inflammatory cytokine responses in macrophages [9]. This inhibition was subsequently associated with bacterial membrane-derived lipids [10]. However, since much of this data was generated using *in vitro* infection of cultured macrophages or dendritic cells (DC), and often at very high multiplicity of *F. tularensis* exposure, its relevance to biologically relevant air-borne Type A *F. tularensis in vivo* infection is unclear as these infections are characterized by relatively low exposure dose and involvement of multiple cell types present in the lung. Furthermore, while the ability to replicate within macrophages is generally associated with virulence [11], *F. tularensis* mutants deficient in intramacrophage replication are not attenuated for virulence in the murine model of pneumonic tularemia [12], suggesting that pathogenesis is not exclusively linked to the bacteria’s ability to antagonize macrophage activation and that involvement and infection of other cell types is also important.

Measuring changes in the expression levels of cellular genes is a powerful tool to study pathogen-host interactions and can yield important insights into how host cells recognize bacteria and how bacteria manipulate host biological processes to facilitate their own replication/dissemination. While studies have utilized transcriptional profiling to characterize the absence of innate immune gene expression during acute *Francisella* infection both *in vitro* [13–15] and *in vivo* [16–19], none have investigated the extent to which this is due to evasion of or active antagonism of host defense response pathways. An understanding of which inflammatory pathways are targeted specifically by highly virulent *F. tularensis* will provide valuable information about bacterial pathogenesis as well as provide insight into the complex regulatory networks governing inflammatory responses during bacterial infection.

Toll-like receptor 4(TLR4) plays a key role in detecting the presence of Gram (−) bacteria through recognition of lipopolysaccharide (LPS) present on the surface of the bacteria, initiating signaling cascades that culminate in pro-inflammatory and interferon-inducible gene expression. Previous studies have shown that infiltration of monocytes and activation of DCs in the lungs of mice exposed to an aerosolized LPS is partially inhibited by prior infection with Type A *F. tularensis* [3, 6] suggesting that the bacteria is actively antagonizing at least some part of the response to TLR4 engagement and subsequent response. *E. coli* LPS-induced production of IL-12B was also found to be partially suppressed in human dendritic cells treated with membrane—derived lipids from virulent, but not attenuated, *Francisella*, although this required relatively high concentrations (30 ug/ml) of lipids [10]. While inhibition of NF-kβ, IRF1 (interferon regulatory factor 1) and IRF8 (interferon regulatory factor 8) activity has been linked to suppression of IL-12B production [10], the molecular mechanism involved is not clearly defined and the potential involvement of additional immune-related transcriptional regulators remains unknown. Moreover, these studies examined the suppression of a limited number of inflammatory mediators and so the true extent of *Francisella*’s ability to antagonize TLR4 signaling is not known.

Stimulation of TLR signaling culminates in the induced expression of hundreds of genes [20–22]. Understanding which genes are specifically targeted by *Francisella* will further help define the extent and molecular mechanisms of immune suppression. In the current study, the ability of different *Francisella* strains (*F. tularensis* subsp. *tularensis* SchuS4*,* Type B *F. tularensis* subsp *holarctica* live vaccine strain (LVS), or *F.novicida*) to modulate the pulmonary transcriptional response to a TLR4 agonist was investigated using an aerosol exposure mouse model. This provides a detailed molecular portrait of the transcriptional response to a TLR4 agonist *in vivo* to more clearly define the scope of modulation and identify inflammatory genes/pathways that are specifically antagonized by a virulent *F. tularensis* infection.

## Methods

### Bacteria

As described previously [23], *Francisella tularensis* subspecies *tularensis* SchuS4 (CDC, Fort Collins, CO) was grown to stationary phase with agitation at 37 °C in Mueller Hinton broth supplemented with 2 % Isovitalex (BBL), pelleted, suspended in PBS with 20 % glycerol, aliquoted, and stored at −80 °C. The post-freeze titer of this stock was 3 × 10^9^ CFU/ml when cultured on cysteine heart agar supplemented with 2 % hemoglobin. *Francisella tularensis* subspecies *holarctica* live vaccine strain (LVS) was obtained from Karen Elkins (FDA, Rockville, MD). Bacteria were grown to stationary phase in Mueller Hinton broth supplemented with 2 % Isovitalex, 1 % glucose, and 0.25 % ferric pyrophosphate, washed, suspended in PBS with 20 % glycerol aliquoted and stored at −80 °C as previously described [23]. The post-freeze titer of this stock was 4 × 10^9^ CFU/ml when cultured on modified Mueller Hinton agar supplemented with 2 % hemoglobin and 1 % Isovitalex. *F. novicida* U112 strain (Francis Nano, University of Victoria, Canada) was grown to stationary phase with agitation at 37 °C in tryptic soy broth supplemented with 01 % L-cysteine, diluted in 20 % glycerol, aliquoted, and stored at −80 °C. The post-freeze titer of this stock was 2 × 10^9^ CFU/ml when cultured on tryptic soy agar supplemented with 0.1 % L-cysteine.

### Mice

Male and female BALB/c and C57BL/6 mice 8–10 weeks of age and free of specific pathogens were purchased from Jackson Laboratories (Bar Harbor, ME). Mice with targeted deletions of myeloid differentiation response 88 (MyD88^−/−^) were kindly provided by Adeline Hajjar at the University of Washington [57] This line was originally obtained from Shizuo Akira (Osaka, Japan) and had been backcrossed at least 6 generations to C57BL/6 mice [58]. Mice with targeted deletions of Toll/interleukin-1 receptor domain-containing adaptor inducing interferon beta (TRIF^−/−^) also were originally obtained from S. Akira and were backcrossed at least six generations to C57BL/6 mice [59]. Mice were housed in laminar flow cages and permitted ad lib access to sterile food and water as previously described [23]. Animal studies were conducted in compliance with the National Research Council Guide for the Care and Use of Laboratory Animals. The mouse experiments were specifically approved by the University of Washington Institutional Animal Care and Use Committee, under protocol numbers 2671–06 and 2982–03.

### Mouse exposures and tissue harvests

Cohorts of mice (three to four animals/group) were exposed to aerosolized bacteria in a whole animal exposure chamber with a computer interface to control pressures and flows (Biaera Technologies, Frederick MD). As described previously [23], aerosols were generated by mini-Heart nebulizers with a flow rate of 8 L/min at 40 psi. Dilution air was regulated at 11.5 L/min to maintain total chamber flow at 19.5 L/min during a 10-minute exposure. Aliquots of frozen bacteria stock (*F. tularensis* subspecies *tularensis* (FT SchuS4), *F. tularensis* subsp. *holarctica* (FT LVS), or *F. novicida* (FN)) were thawed and diluted 1:4 in PBS for nebulization, targeting a lethal dose for each strain. Actual bacterial deposition in the lungs in each experiment was determined by quantitative culture of homogenized lung tissue harvested from three sentinel mice euthanized immediately after aerosol exposure. Measured depositions for each pathogen were as follows: *F. tularensis* subsp. *tularensis* (SchuS4) (242, 809, 74 CFU/lung), *F. novicida* (2827, 2673, 3805 CFU/lung), *F. tularensis* subsp. *holarctica* (FT LVS) (1580, 1356, 1538 CFU/lung). Survival studies demonstrated that the LD50s for aerosol exposure is less than 200 CFU/lung for FT SchuS4 and FN (Skerrett, unpublished). The reported LD50 for FT LVS in Balb/c mice infected by the aerosol route is approximately 1000 CFU/lung [60, 61]. Mock-infected control mice were exposed to aerosolized PBS. To determine the effect of *Francisella* infection on responsiveness to LPS, mice were exposed to aerosolized *E. coli* 0111:B4 LPS (Ultrapure, List) at a concentration of 100 μg/ml 18 h after inhalation of FT SchuS4, FT LVS, FN or PBS. After 2.5 h from the onset of LPS exposure, mice were euthanized with pentobarbital and exsanguinated by cardiac puncture. The pulmonary arteries were perfused with 5 ml cold PBS and the lungs homogenized in Qiazol extraction solution. The infections and subsequent LPS exposures were conducted once (3–4 animals/group) for each *Francisella* strain.

### RNA isolation and expression microarray analysis

Total RNA was isolated from lung by homogenization (10 % w/v) in Trizol (Invitrogen, Carlsbad, CA) followed by chloroform extraction and isopropanol precipitation as previously described [23] and RNA quality assessed using a BioAnalyzer (Agilent Technologies, CA). Gene expression profiling experiments using Agilent Mouse Whole Genome 44 K microarrays and data processing/analysis was performed as previously described [23]. Briefly, fluorescent probes were prepared using Agilent QuickAmp Labeling Kit according to the manufacturer’s instructions. Spot quantitation was performed using Agilent’s Feature Extractor software and all data entered into a custom-designed database, SLIMarray (http://slimarray.systemsbiology.net), and then uploaded into Genedata Analyst 8.0 (Genedata, Basel, Switzerland). Data normalization was performed in Genedata Analyst using central tendency followed by relative normalization using pooled RNA from mock infected mouse lung (*n* = 6) as reference. Transcripts differentially expressed (at least 2-fold, *p* value < 0.01) between infected and control animals were identified by standard *t* test using the Benjamini-Hochberg procedure to correct for false positive rate in multiple comparisons. Ingenuity Pathway Analysis and Entrez Gene (www.ncbi.nlm.nih.gov/sites) were used for mammalian gene ontology and pathway analysis.

### Availability of data and materials

The complete microarray dataset has been deposited in NCBI’s Gene Expression Omnibus [62] and is accessible through GEO Series accession number GSE65871.

### Quantitative RT-PCR

Quantitative real-time PCR was used to estimate *Francisella* loads in lung tissue as previously described [23]. Reverse transcription of total lung RNA was performed using either gene-specific primers or random primer and the Superscript III First Strand cDNA synthesis kit (Invitrogen, Carlsbad, CA). Primers and probes used for detection of *Francisella* were taken from [63].

## Results

### Active suppression of the TLR4 signaling pathway is specific to virulent Type A *F. tularensis*

BALB/c mice were first exposed to either aerosolized PBS, *Francisella tularensis* subsp *tularensis* (FT SchuS4), the live vaccine strain of *Francisella tularensis* subsp. *holarctica* (FT LVS), or *Francisella novicida* (FN). Eighteen hours after exposure to aerosolized bacteria or PBS, infected and control animals were exposed to aerosolized *E. coli* LPS, a potent TLR4 agonist. This time-point was chosen based on a previous study showing a lack of induction of immune-related gene expression in the initial 24 h post-exposure [23]. Lung samples were harvested 2.5 h after LPS exposure to measure changes in the host transcriptome using microarrays.

**Fig. 1:**
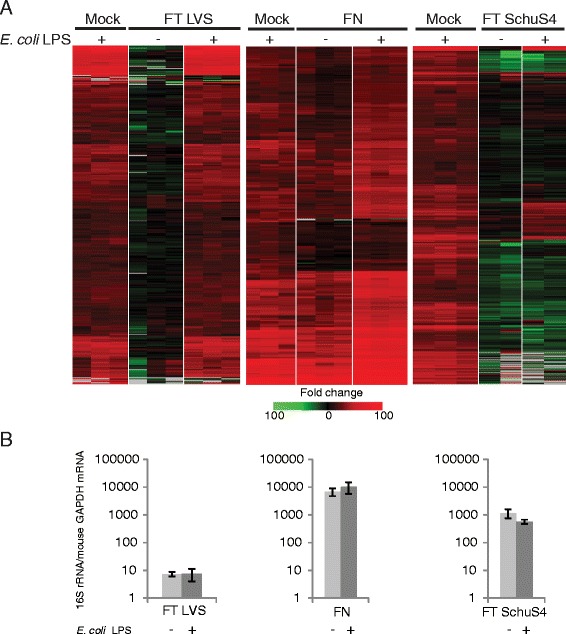
*Francisella*-mediated antagonism of TLR4-mediated signaling correlates with strain virulence and not bacterial replication levels. **a** Heatmap represents expression profiles of sequences that are regulated (at least 2-fold change in median expression level, *p* value < 0.01) in lung tissue in response to aerosolized *E. coli* LPS. Each panel reflects separate exposure studies with BALB/c mice infected with either *F novicida* (FN), *F. tularensis* subsp, *holartica* (LVS) or *F. tularensis* subsp. *tularensis* (SchuS4). Each column represents gene expression data comparing RNA from lung tissue from an individual infected animal to pooled RNA isolated from mock animals (*n* = 6). Sequences shown in red represent increased expression. Sequences shown in green represent decreased expression and sequences shown in black indicate no change in expression in treated relative to control animals. **b** Replication levels of *Francisella* strains in lungs of Balb/c mice approximately 20.5 h post-infection as measured by qPCR of 16S rRNA. Data represents mean and standard deviation of three individual animals

Each experiment compared 4 groups of mice: mice exposed to aerosolized bacteria alone, mice exposed to aerosolized bacteria followed 18 h later by exposure to aerosolized LPS, mock infected mice exposed to aerosolized PBS followed 18 h later by exposure to aerosolized LPS, and mock infected control mice exposed to aerosolized PBS alone. As shown in Fig. 1a, exposure of mock-infected animals to aerosolized *E. coli* LPS results in the increased expression (at least 2-fold increase in median expression level, *p* value < 0.01) of up to 1700 genes relative to control animals exposed to aerosolized PBS alone, depending on the exposure. Very little change in *E. coli* LPS-responsive genes occurred in the lungs of animals exposed to either FT LVS (Fig. 1a, *left panel*) or FT SchuS4 (Fig. 1a, *right panel*) alone. Animals exposed to FN alone showed increased expression of the majority of LPS-responsive genes, although generally at a lesser degree of change than observed in *E. coli* LPS-exposed control animals (Fig. 1a, *middle panel*). Purified *F. novicida* LPS neither stimulates nor antagonizes signaling through murine TLR4, suggesting that it does not directly interact with this receptor [24]. Furthermore, inhalation of *F. novicida* LPS does not elicit an inflammatory response in vivo [24]. Therefore, induction of LPS-responsive genes in FN-infected lung tissue is unlikely to be mediated by FN LPS engagement of TLR4.

Subsequent exposure of both FT LVS and FN-infected animals to *E. coli* LPS resulted in increased expression of LPS-responsive genes to levels generally similar to that seen in control animals exposed to *E. coli* LPS (Fig. 1, *left and middle panel*, respectively), suggesting that these strains do not actively antagonize TLR4 signaling. Indeed, the expression level of these genes in FN-infected, *E.coli* LPS-exposed animals were generally higher than the animals exposed to *E. coli* LPS alone. In contrast, exposure of FT SchuS4-infected animals to *E. coli* LPS did not result in extensive change in expression levels of LPS-responsive genes (Fig. 1a, *right panel*). The majority showed either no increase in expression or did not increase to levels comparable to those observed in LPS-treated control animals. Suppression of LPS-induced gene expression during FT SchuS4 infection is unlikely to be directly related to bacterial burden per se. FT SchuS4 and FT FN replicate rapidly in murine lungs and FN was present at higher concentrations than FT SchuS4 at the time of analysis (Fig. 1b) and yet did not significantly impact the transcriptional response to *E. coli* LPS. Furthermore, animals were exposed to a higher dose of FT FN than FT SchuS4 (approximately 15 LD50s vs 5 LD50s, respectively). It is possible that the lack of inhibition in the presence of FT LVS infection is partly due to the lower level of replication in the lungs of animals infected with this strain, resulting in a bacterial burden that was approximately 100-fold lower than that of FT SchuS4 (Fig. 1b), consistent with a previous study [23]. Exposure of infected animals to *E. coli* LPS did not appear to significantly impact the replication level of any of the *Francisella* strains. This data provides further molecular evidence of a previous report demonstrating lack of immune cell infiltration in the lungs *F. tularensis*-infected animals challenged with *E. coli* LPS [3].

### *Francisella* targets signaling pathway downstream of TLR4 molecule

**Fig. 2:**
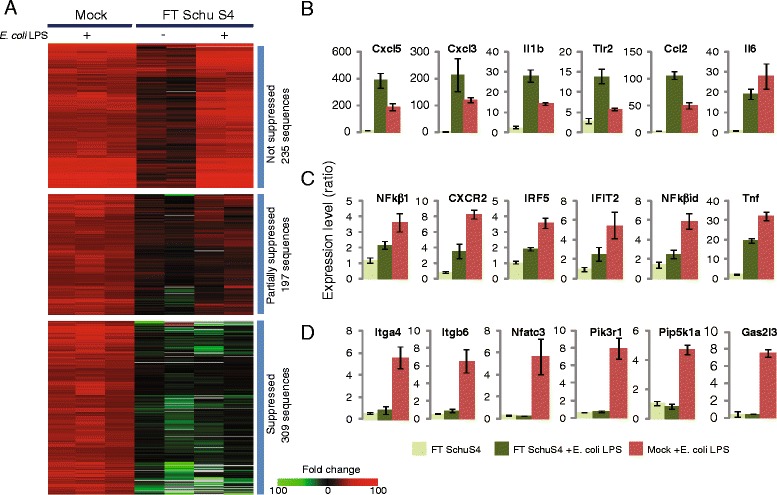
Differential effect of *F. tularensis* SchuS4 infection on *E. coli* LPS-responsive gene expression. **a** Expression profiles of LPS-responsive sequences that are not suppressed (upper panel), partially suppressed (middle panel) or suppressed (lower panel) by prior FT Schu4 infection. Each column represents gene expression data from an individual experiment comparing RNA from lung tissue from an infected animal to pooled RNA isolated from mock animals (*n* = 6). Sequences shown in red represent increased expression, sequences shown in green represent decreased expression and sequences in black indicate no change in expression in treated relative to control animals. Bar graphs represent expression levels (measured by microarray) of individual LPS-responsive genes that were not suppressed (**b**) partially suppressed (**c**) or suppressed (**d**) by prior FT SchuS4 infection. Data represents mean fold-change and standard deviation of two or three individual animals relative to control animals. Only sequences that were 3-fold induced (*p* value < 0.01) in all three LPS-exposed control animals (*n* = 978) were used for this analysis

K-means cluster analysis of the expression profiles of *E.coli* LPS-inducible transcripts in lung tissue from control, FT SchuS4 infected and FT SchuS4-infected mice challenged with *E. coli* LPS revealed three main groups with distinct expression patterns. The first group included sequences that were either not induced or slightly induced (<3-fold) in FT SchuS4-infected alone but highly induced in FT SchuS4-infected animals subsequently challenged with *E. coli* LPS (Fig. 2a*upper panel* and b, and Additional file 1: Table S1). The expression level of these sequences was either equivalent or even higher than that observed in mock animals challenged with *E.coli* LPS and so were designated “not suppressed” by FT SchuS4. The second group included sequences that were either not induced or not significantly (<3-fold) induced in FT SchuS4 alone but were more highly induced in FT SchuS4-infected animals subsequently challenged with *E. coli* LPS (Fig. 2a*middle panel* and c, Additional file 2: Table S2). While exposure of FT SchuS4-infected animals to *E. coli* LPS did induce expression of these sequences to a level generally higher than was observed in FT SchuS4-infected animals, the levels were generally still lower relative to *E.coli* LPS-exposed control animals and so these transcripts were designated “partially suppressed”. Lastly, transcripts that were not induced in either FT SchuS4 or FT SchuS4-infected animals challenged with *E. coli* LPS (Fig. 2a*lower panel* and d, Additional file 3: Table S3) were designated “suppressed”.

Overall, approximately two thirds of *E. coli* LPS-inducible transcripts were either partially or completely suppressed by prior *Francisella* infection, suggesting significant antagonism of TLR4 signaling. However, the differential effect on LPS-responsive gene expression suggests that FT SchuS4 is not targeting the TLR4 molecule directly but rather downstream in the signaling cascade, such as an adaptor protein(s) or transcription factor(s). One possibility is that *Francisella* is targeting either the MyD88—dependent or MyD88-independent (TRIF-dependent) arms of the TLR4 pathway, which each result in the activation of specific transcription factors [20, 21]. To define the contribution of each adaptor protein in LPS-induced gene expression in the lungs, groups of four wild-type, MyD88^−^/^−^ and TRIF^−^/^−^ C57BL/6 mice were exposed to aerosolized *E. coli* LPS or aerosolized PBS. For each microarray experiment, RNA from *E. coli* LPS-treated animals was compared with RNA from the PBS-mice of the same genetic line (e.g.. TRIF^−^/^−^ after aerosolized LPS versus TRIF^−^/^−^ after aerosolized PBS). Sequences showing increased expression in wild-type mice exposed to *E. coli* LPS relative to PBS-treated were used as a reference to identify a comprehensive list of LPS-responsive genes in the C57BL/6 background. The profiles of these sequences were then assessed in each of the knockout strains to define MyD88 and TRIF-dependent gene expression. The absence of the MyD88 adaptor protein exhibited a much greater impact on expression of LPS-responsive sequences than TRIF, as evidenced by the absence or reduced expression of a large number of sequences in the MyD88^−^/^−^ animals compared to wild-type animals (Additional file 4: Figure S1A). This is consistent with MyD88 being particularly important for initiating host response to Gram-negative bacterial infections [25]. A much smaller number of LPS-responsive genes were dependent on the presence of TRIF, as shown by their lack of expression in TRIF^−^/^−^ mice (Additional file 4: Figure S1A).

The expression profiles of LPS-responsive sequences that were not suppressed, partially suppressed or suppressed by *Francisella* infection were then assessed in each of the knockout strains. For this analysis, only LPS-responsive genes (*n* = 471) commonly induced in both BALB/c and C57BL/6 mice were used due to differences in the transcriptional response to *E. coli* LPS between these two strains (data not shown). Most LPS-inducible genes not affected by *Francisella* infection did not show increased expression in the MyD88^−^/^−^ mice, indicating that their expression is dependent on the presence of MyD88 (Additional file 4: Figure S1B). Similarly, the majority of LPS-responsive genes that were partially or completely suppressed by FT SchuS4 were also dependent on MyD88. None of the sequences appeared to be completely dependent on the presence of TRIF, as evidenced by their induction in TRIF ^−^/^−^ mice exposed to *E. coli* LPS. This is likely due to the small number of TRIF-dependent genes in general, making it difficult to assess the role this adaptor molecule may play in *Francisella*-mediated suppression. Collectively, these data demonstrate attenuation of only a subset of MyD88-dependent genes in the presence of *Francisella* infection, making it unlikely that MyD88 is directly targeted in which case all MyD88-dependent genes would be expected to be affected.

### Upstream analysis of *E. coli* LPS-responsive gene expression identifies potential targets antagonized by *F. tularensis* infection

**Fig. 3:**
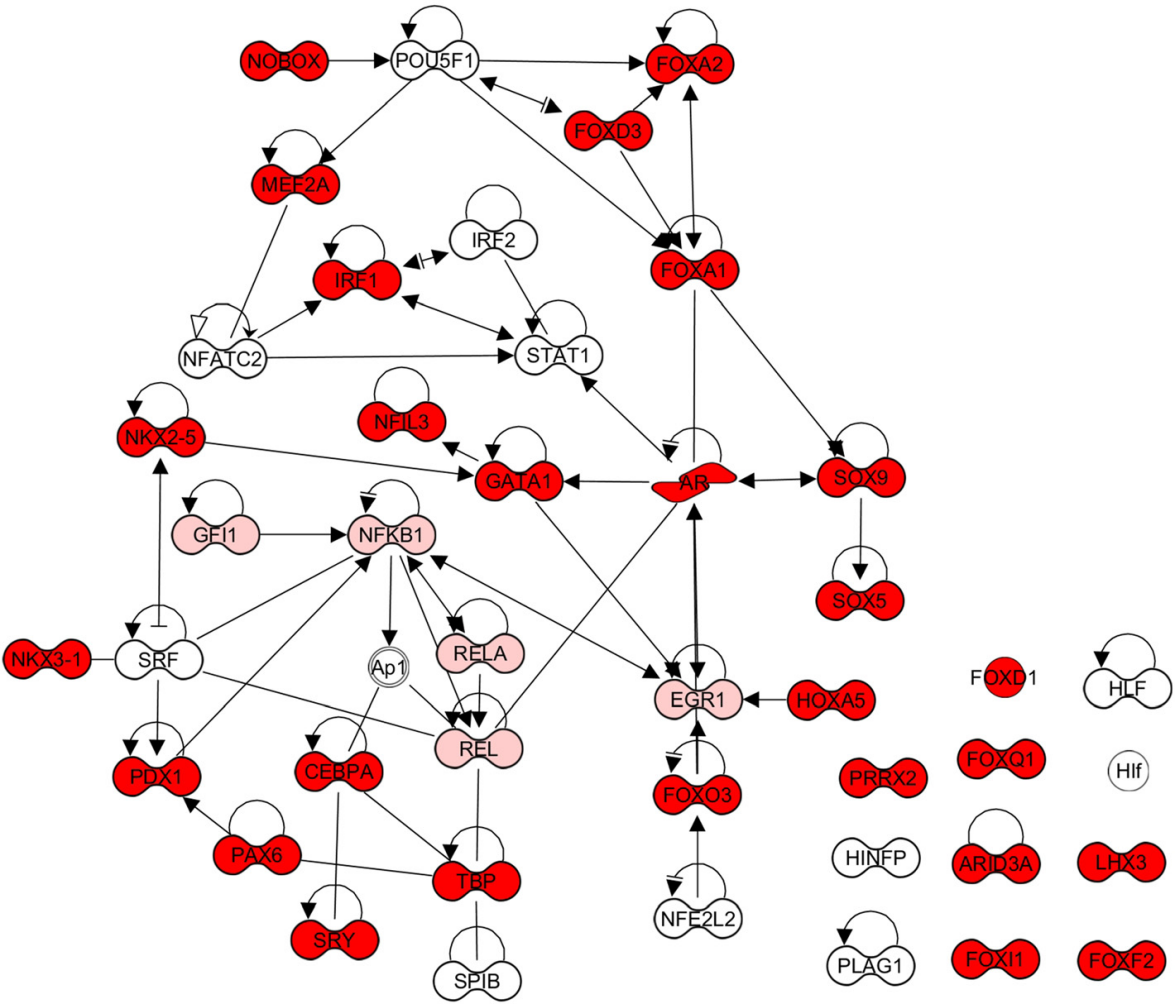
Network of transcription factors predicted to regulate *E. coli* LPS-responsive gene expression based on overrepresentation of binding sites. TFs with binding sites overrepresented in genes altered (suppressed or partially suppressed) by *F. tularensis* SchuS4 infection are indicated in red and pink, respectively. Lines indicate direct interaction: For visualization, not all interactions between TFs are shown

oPOSSUM, a web-based system for the detection of over-represented conserved transcription factor (TF) binding sites in sets of genes [26, 27], was used to identify candidate transcription regulators that may be targeted by *Francisella* based on the expression profiles of their target genes. The results of the oPOSSUM analysis of the 972 genes showing increased expression in lung tissue from animals exposed to aerosolized *E. coli* LPS showed significant enrichment of the binding sites for 43 TFs (Table 1 and Fig. 3). These included key TFs known to be activated by the TLR4 signaling cascade (AP1, IRF1 and 2, NFκβ1, Rel, RelA, Sp1, Stat1) in addition to numerous factors not previously associated with this pathway.

**Table 1:** oPOSSUM 3.0 results for LPS-induced genes suppressed, not suppressed or partially suppressed by *F. tularenesis*

		All LPS-induced genes			Genes suppressed			Genes not suppressed			Genes partially suppressed		
TF	Family	Z-score	Fischer score	*q*-value	Z-score	Fisher score	*q*-value	Z-score	Fisher score	*q*-value	Z-score	Fisher score	*q*-value
AP1	Leucine Zipper	5.492	49.37	0.0004501									
Ar	Hormone-nuclear Receptor	9.599	8.988	0	7.618	7.526	5.36E-10						
ARID3A	Arid	18.069	53.054	0	20.009	38.878	0				6.948	15.813	2.65E-07
CEBPA	Leucine Zipper	17.508	56.795	0	7.313	24.871	5.02E-09				7.727	19.6	1.11E-09
Egr1	BetaBetaAlph-zinc finger	9.242	35.76	0							10.597	15.075	0
FOXA1	Forkhead	9.272	46.98	0	9.751	29.455	0						
Foxa2	Forkhead	7.135	47.333	1.38E-08	12.479	41.802	0						
FOXD1	Forkhead	13.729	53.842	0	14.463	39.964	0						
Foxd3	Forkhead	17.779	49.257	0	15.734	40.563	0						
FOXF2	Forkhead	6.49	20.958	1.10E-06	7.19	21.679	1.20E-08						
FOXI2	Forkhead	14.135	36.86	0	16.058	36.651	0						
FOXO3	Forkhead	14.321	50.651	0	12.83	37.983	0				5.324	14.497	0.003402
Foxq1	Forkhead	10.523	44.105	0	11.804	42.968	0						
Gatal	GATA	9.843	45.572	0	6.597	31.379	7.22E-07						0.013187
Gfi	BetaBetaAlpha-zinc finger	12.107	48.885	0							7.9	15.946	3.61E-10
HLF	Leucine Zipper	11.858	24.551	0									
HOXAS	Homeo	19.619	55.444	0	20.668	35.237	0				5.306	16.325	0.003501
IRFI	IRF	16.593	37.42	0	11.588	30.497	0				6.563	11.061	2.93E-06
IRF2	IRF	10.478	10.828	0									
Lhx3	Homeo	10.204	21.206	0	15.823	27.512	0						
MEF2A	MADS	11.261	28.869	0	5.667	22.212	0.000234				5.164	9.596	0.007108
MIZF	BetaBetaAlpha-zinc finger	5.96	15.897	3.14E-05	8.243	20.427	4.82E-12						
NFATC2	Rel	7.841	54.491	6.71E-11				7.61	8.397	1.70E-09			
NFE2L2	Leucine Zipper	5.901	27.696	4.39E-05									
NFIL3	Leucine Zipper	14.295	38.826	0	9.437	35.141	0						
NF-kappaB	Rel	13.665	56.778	0				39.032	14.744	0	5.492	16.553	0.001656
NFKB1	Rel	5.708	14.056	0.0001325									
Nkx2-5	Homeo	22.118	58.941	0	26.88	44.266	0						
NKX3-1	Homeo	15.244	41.36	0	17.48	37.027	0						
Nobox	Homeo	9.798	46.489	0	12.618	43.858	0						
Pax6	Homeo	6.502	10.168	1.04E-06	10.422	16.405	0						
Pdx1	Homeo	13.632	51.723	0	19.67	41.917	0						
PLAG1	BetaBetaAlpha-zinc finger	7.702	14.764	1.95E-10									
Pou5fl	Homeo	6.767	12.53	1.77E-07									
Pnx2	Homeo	14.121	49.554	0	18.221	38.381	0						
REL	Rel	11.771	55.54	0				30.632	10.072	0	5.323	11.424	0.003402
RELA	Rel	13.26	45.068	0				42.057	16.996	0	5.945	9.196	0.000138
Sox5	High Mobility Group	15.026	49.598	0	18.695	46.646	0				5.001	15.29	0.013577
SOX9	High Mobility Group	8.54	45.725	0	12.388	30.748	0						
SPIB	Ets	5.794	74.595	8.15E-05				9.177	7.168	0			
SRF	MADS	8.106	8.71	6.92E-12									
STAT1	Stat	6.86	30.655	9.53E-08				20.574	7.325	0			
TBP	TATA-binding	14.562	52.455	0	11.841	43.297	0				7.908	14.859	3.61E-10

Background: All 29,347 genes in oPOSSUM database, Conservation: 0.4, Matric Threshold: 85 %, Search region +/−2000 bp. *Q*-value represents FDR adjusted *p*-values

Binding sites for 27 of these 43 TFs were also over-represented in LPS-responsive genes suppressed by *Francisella* infection, suggesting that their activity may be perturbed in the presence of bacteria (Table 1 and Fig. 3). This included numerous members of the forkhead family of TFs, implicated in a wide range of cellular functions including modulating inflammatory responses by antagonizing pro-inflammatory transcriptional activities (FoxA2, FoxD1, FoxO3), lymphoid homeostasis (FoxO3), immunosuppressive properties of T regulatory cells (FoxA1), suppressing Th2 immunity (FoxA2), NK cell function (Foxq1) and acute lung injury (FoxA1). Since many of these TFs function to repress transcription of their target genes, the binding site enrichment may actually indicate activation by *Francisella*. In support of this, the expression levels of Foxf2, Foxa2, Foxd1 and Foxd3 were all increased in FT SchuS4 and FT SchuS4 + *E.coli* LPS treated mice, but not in *E. coli* LPS-treated control animals, nor were they induced in FN or LVS-infected animals exposed to *E. coli* LPS (data not shown). The binding site for IRF1 was also over-represented in this group, consistent with a previous report showing inhibition of nuclear translocation of this TF by *Francisella* [10]. Other candidate regulators included numerous TF involved in immune cell differentiation/proliferation (NFIl3, Sox5, Sox9, Plag1, CEBPA, Gata1, Arid3A), immune cell infiltration (Pax6, Hoxa5) and dampening inflammation (Prrx2, Pdx1).

Interestingly, binding sites for NfKappaB, Rel and RelA, part of the key TF complex activated via MyD88 signaling, were overrepresented only in sequences that were either not suppressed or partially suppressed by *Francisella* infection (Table 1). This is consistent with the data showing that many MyD88-dependent genes are not suppressed during infection and suggests the bacteria only partially blocks activation of this important inflammatory transcription complex. Binding sites for STAT1, SP1B, and NFATC2 were also overrepresented in LPS-responsive gene expression unaffected by the presence of *Francisella*. This analysis suggests that *Francisella* may not significantly impact the activity of the main transcription factors known to be activated as a result of TLR4 engagement and subsequent signaling cascade. However, it identified numerous other potential candidate TF, possibly functioning downstream of the immediate TLR4 signaling cascade, that may be perturbed by *Francisella* infection to mediate antagonism of host immune responses.

### Expression of key inflammatory mediators is not antagonized by *Francisella*

**Fig. 4:**
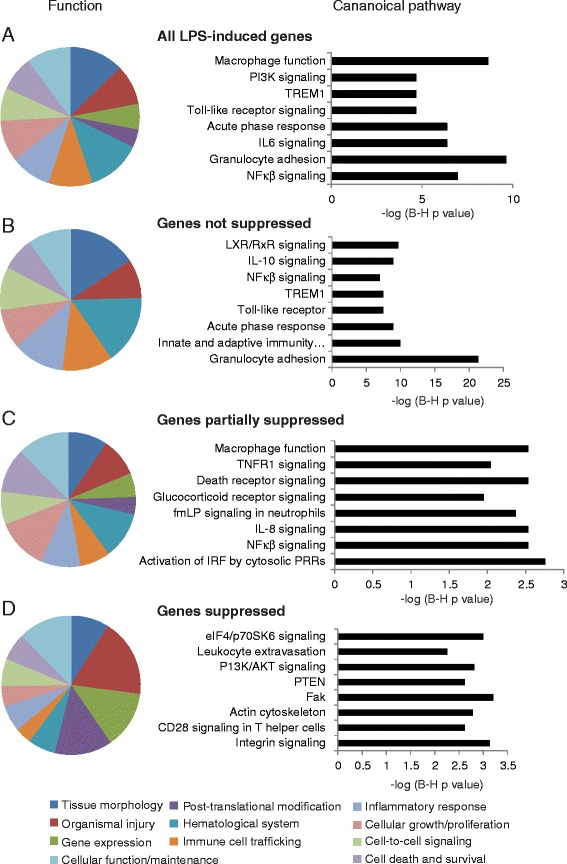
Functional annotation of *E. coli* LPS-responsive gene expression in the absence and presence of *F. tularensis* SchuS4 infection. Top scoring functional categories and canonical pathways are shown for all LPS-inducible genes (**a**) LPS-inducible genes not suppressed (**b**) partially suppressed (**c**) or suppressed (**d**) by *Francisella* infection

To gain insight into which LPS-associated cellular processes are perturbed by FT SchuS4, gene ontology analysis of LPS-responsive genes that were i) not suppressed, ii) partially suppressed or iii) suppressed by *Francisella* infection was performed. As expected, analysis of all LPS-inducible sequences (*n* = 978) showed enrichment for genes predominantly involved in host defense responses, including hematological system, immune cell trafficking, inflammatory response, lymphoid tissue structure and infectious disease (Fig. 4a). Enriched canonical pathways included classic pro-inflammatory signaling such as NF-κβ, IL6 and TLR pathways (Fig. 4a).

Surprisingly, LPS-responsive sequences not suppressed by *Francisella* infection were also predominately enriched for genes related to classic pro-inflammatory signaling (Fig. 4b). This included numerous genes associated with NFκβ/TLR signaling (NFkb2, Nfkbia, Nfkbie, NFkbiz, Nod2, RelB, Map3k8, MyD88, Ripk2, Irak3, Tifa) as well as the majority of pro-inflammatory chemokines/cytokines induced by *E.coli* LPS (including Ccl2, Ccl3, Ccl4, Ccl7, Ccl9, Ccl17, Ccl19, IL1a, IL1b, Cxcl3, Cxcl5, Cxcl9). While this is consistent with the enrichment of NF-κβ TF binding sites in this gene set, it is intriguing that these genes are not antagonized, at least at the level of transcription, as they play a key role in both recruiting and activating immune cells critical for successful clearance of *Francisella*. Many genes induced by inflammatory cytokines (SelE, Saa1, Saa2, Vacm1, Vnn3, Socs3) were also not suppressed, suggesting that endothelial/epithelial cells are responding to actions of these cytokines. The lack of suppression of genes known to play a role in dampening inflammation (Zc3hl2a, Irg1, Irak3, Il1r2, Il1rn, Tyrobp, Lilb4) suggests the bacterium may be utilizing host pathways to suppress immune responses. Targeting host processes responsible for regulating immune homeostasis is an effective way of preventing host responses that are detrimental to the survival of pathogens. Indeed, Irg1 inhibits TLR-triggered production of IL-6 and TNFα, both of which are the only two pro-inflammatory cytokines that are partially suppressed during infection.

### Perturbation of innate antiviral defense responses, gene transcription, intracellular trafficking and actin cytoskeleton by *Francisella* infection

LPS-response sequences that are partially suppressed by *Francisella* infection were also enriched for genes which function in directly in mediating inflammation but the specific functions were generally different than those unaffected by *Francisella* (Fig. 4c). Many are involved in innate antiviral responses (Ifi203, Ifih1, Ifitm2, Ifitm6, Ifngr2, IRF5, Ddx58, Ddx60, Trim6, Trim56, lamp3, Trim30d). Although only two prominent inflammatory cytokines (IL6 and TNFα), two chemokines (Cxcl1, Cxcl2) and TFs (Mef2a, Stat3, Nfκβ-1, IRF5, Nfκβid) were partially suppressed, there were many genes related to activation of IL8 signaling, including the receptor Cxcr2. While LPS-induced expression of mouse IL8 homologues Cxcl1/Cxcl2 [28] were partially suppressed, they were still very highly induced in FT SchuS4-infected animals (>100-fold). The other IL8 homologue, CXCL5, was induced to extremely high levels (approximately 400-fold) in FT SchuS4-LPS-expsed animals, compared to approximately 200-fold in LPS-exposed control animals. The IL8 pathway can trigger neutrophil chemotaxis, respiratory burst, granule release and also increase adhesion [29]. Partial suppression of genes encoding proteins involved in differentiation/function of macrophages/monocytes (Cx3cr1, Lyz2) and neutrophils (Lcn2, Ly6a, Lyz2, Nab1) indicate the bacterium may be antagonizing these immune cells in mechanisms independent of blocking their recruitment. Others were associated with glucocorticoid receptor signaling (such as Nr3c1, Ptgs2, Creb, Pik3c2a, Pik3R1,Gmeb2), which also includes genes from NFkappaB (NF-κβ1, Ascc1, Erc1, Map3k2) and TNF signaling (Tnfrsf11a, TNF-α). Activation of this pathway can either repress transcription of inflammatory genes via inhibition of AP1, NF-κβ and NF-AT nuclear translocation, or induce anti-inflammatory gene expression.

Interestingly, many genes partially suppressed by *Francisella* infection are associated with intracellular trafficking, including multiple Rab proteins (Rab1, Rab11a, Rab11fip2, Rab28). The Rab11a GTPase controls TLR4-induced activation of IRF3 [30] which may contribute to the reduced expression of numerous IFN-induced sequences observed in infected mice. Genes essential for trafficking of lysosomal enzymes (Gnptab, Trim23, Trim30d, Vma21), transport of cargo through the golgi apparatus (Golim4, Golph3, Vps35, Synrg), endoplasmic reticulum (Sec13, Sec22a, Sec63) and general intracellular trafficking (Arcn1, Exosc1) were also modulated in presence of infection. As an intracellular pathogen, *Francisella* is known to modulate intracellular trafficking, including impairment of phagosome maturation and disrupting the vesicle membrane to escape into cytosol [31].

Gene ontology analysis of LPS-responsive gene expression completely inhibited by *Francisella* infection showed greater representation of fundamental cellular processes such as gene expression, post-translational modification, and cell injury (Fig. 4d). Gene expression-related processes involved chromatin modification, including histones (Hist1hle, Hist1h2ab, Hist1h4d, Hist1h4f, Hist2h4, Hist4h4) and chromodomain helicases (Chd1, Chd4, Chd6, Chd9), which controls access of the transcription complex to DNA. The expression levels of genes involved in mRNA processing, such as editing/splicing (Celf2, Cpeb4, Ddx23, Prpf4og, U2af2, Sf3b1, Tra2b), stability (Csdel, Ddx6, Smg1) and export (Hnrnpao, Hnrnpr) were also significantly decreased in the presence of infection, as were many genes involved in transcription initiation. This may explain the global decrease in cellular gene expression observed during acute *F. tularensis* infection [23]. Inhibition of numerous genes related to ubiquitin modification of proteins, including E3 ubiquitin-ligases (Cbll1, Hecd1, Huwel, Urb2, Urb3), ubiquitin-conjugating enzymes (Ube2k, Ube2w) and ubiquitin-specific peptidases (Usp15, Usp47) suggest post-translational modification mechanisms is also an important target of *F. tularensis*.

LPS-responsive gene expression associated with cell growth/proliferation also appeared to be particularly targeted by *F. tularensis*, including those associated with the eIF4/p70 SK6 pathway (eIF2, eIF4EBP2, Itga4, PPP2R3A, Rn18) which activates the PI3K/AKT pathways in response to growth factors and cytokines. Proliferation-related genes included genes which function to both inhibit and promote cell growth, making it difficult to interpret the overall functional effect of this modulation. The P13K/AKT signaling pathways were also targeted by *F. tularensis*, with the expression levels of Pik3c2a, Pik3cb, Pik3r1, Rapgapa2, Prex2, PTEN, ccdc88a and Spnb2 all significantly decreased. Multiple studies have implicated the PI3K/Akt pathway in *F.tularensis*-mediated suppression of cytokine production, although results suggest both activation and repression of the pathway occurs [7, 14, 32, 33].

Genes associated with intracellular trafficking, including clathrin-mediated enodocytosis (Aak1, Fcho2, Hip1, Itsn2), phagocytosis (Acap2, Apbblip, Fnbp1l, Rab43), golgi vesicle trafficking (Arfgapl, Asap2, Cog5, Scfd1, Atad2b, Lman2), secretory pathway (Pcsk5), receptor internalization/membrane trafficking (Git2) and vesicle acidification (Atp6voa4, Atp6voc) were also targeted by Type A *Francisella*. Indeed, *Francisella* has been shown to prevent acidification of their phagosomes [34]. In addition, inhibition of cytoskeleton (Clasp1, Ldb3, Lrch3, Mical2, Pkp4, Svil) and cell motility/adhesion-related genes (such as Ahnak, Itga2, Itga4, Itgb6, Lgals9, Lpp, Ptprb, Cd226, Svil, Git2) suggest the bacterium is targeting processes responsible for cytoskeleton remodeling and migration of cells.

Collectively, while *Francisella* does not appear to directly inhibit expression of canonical inflammatory mediators, it may be targeting cellular processes that collectively could dysregulate an effective innate immune response while at the same time facilitating its own replication.

## Discussion

This study represents the first demonstration that prior infection with Type A *F. tularensis* subsp *tularensis*, not but *F. novicida* or *F. tularensis* subsp *holarctica* LVS, substantially alters the pulmonary transcriptional response to a TLR4 agonist. These results indicate that the absence of host defense response-related gene expression is at least partially attributed to its ability to antagonize TLR4 signaling. It is consistent with and expands upon previous reports demonstrating that Type *A F. tularensis* can attenuate distinct aspects of host inflammatory responses. Specifically, virulent *Francisella* can attenuate on-going cytokine responses to less virulent *F. novicida* in human monocytes [35], partially suppress secretion of TNFα and IL12 in response to TLR2 and TLR4 agonists [6], and inhibit the recruitment of inflammatory cells throughout the lung in response to aerosolized LPS [3]. Recently, lipids from virulent SchuS4, but not attenuated strains, have been found to partially suppress phenotypic maturation of dendritic cells, infiltration of inflammatory cells, and select cytokine secretion [10, 36]. However, the host response to pathogens is both complex and extensive, consisting of both the cell sensing the presence of the pathogen and activating defense pathways and the subsequent modulation of these cellular pathways by the pathogen. Examining the effect of *Francisella* on a limited number of inflammatory mediators may not accurately reflect the overall interplay between the bacteria and innate immune pathways. The current study uses a global approach to more clearly define the scope of perturbation and distinguish between inflammatory genes/pathways that are specifically targeted by *Francisella* versus those that are not.

Due to the challenges of working with highly virulent Type A strains, *F. novicida* and *F*. subsp. *holarctica* LVS are often utilized to investigate the interaction of *Francisella* with innate immune pathways. However, while both of these strains are capable of causing tularemia in mice similar to the most virulent type A strains, they rarely cause serious illness in humans. Their inability to significantly alter the transcriptional response to TLR4 agonists suggests inherent differences in their interaction with immune response pathways. While there is extensive conservation between *Francisella* species at the genome level, regions unique to the virulent Type A strains have been identified [37]. Indeed, important differences in the ability to modulate innate immunity have been demonstrated between virulent and avirulent strains of *Francisella*. For example, while both *F. tularensis* SchuS4 ΔpyrF and LVS ΔpyrF strains are deficient for intramacrophage growth, LVS ΔpyrF induces significantly higher levels of TNF-α than wild-type LVS in exposed macrophages whereas SchuS4 ΔpyrF does not, suggesting it has additional mechanisms for attenuating cytokine expression in macrophages compared to LVS [12]. Induction of MiR-155 by *F. novicida*, but not the virulent Type A SchuS4 strain, has been linked to SHIP down-regulation and enhanced pro-inflammatory cytokine response [38]. TNF-α mediated nuclear translocation of NF-κβ p65 subunit is partially blocked by SchuS4 but not LVS infection [7]. Collectively, these studies demonstrate the importance of utilizing SchuS4 when investigating modulation of inflammatory pathways by virulent *Francisella*.

Surprisingly, although Type A *F. tularensis* subsp. *tularensis* infection significantly altered the host transcriptional response to *E.coli* LPS, mRNA levels of the majority of the classical inflammatory mediators were either unaffected or only partially suppressed. These include genes encoding critical signaling molecules (such as TLR2, MyD88, Map3K8) and transcription factors (JunB, Myc, Fos, NF-κβ). Key inflammatory chemokines responsible for recruiting immune cells, including Ccl2, Ccl9, Cxcl2, Cxcl3, Il1b and numerous others, were induced to either an equivalent level or even higher in *Francisella*-infected animals exposed to *E. coli* LPS relative to similarly treated control animals. The expression of many pro-inflammatory mediators is regulated by MyD88 [21], consistent with data from the current study demonstrating that many MyD88-dependent genes are not actively suppressed by the bacterium. Only expression of TNFα and IL6 was partially inhibited in infected animals, suggesting that the suppression of pro-inflammatory cytokine expression is not as global as previously thought and instead may be restricted to a subset. Interestingly, most inflammatory-related gene expression suppressed (or partially suppressed) by *F. tularensis* is more associated with viral infection and host antiviral responses, including many known to be induced in response to interferon and/or involved in cell entry of numerous viruses. As a predominantly intracellular pathogen, these genes may play a role in as yet undefined anti-bacterial processes.

The lack of pro-inflammatory cytokine gene expression observed during acute *F. tularensis* infection may be more due to lack of recognition as opposed to active targeting of pathways by the bacteria. However, it is also possible that *F. tularensis* utilizes post-transcriptional mechanisms to inhibit cytokine production and subsequent immune cell infiltration. Many of the genes antagonized by *F. tularensis* encode proteins with roles in various intracellular trafficking activities and actin cytoskeleton rearrangement. Intracellular trafficking regulates many aspects of inflammation, including antigen processing, cytokine/TLR receptor trafficking, cytokine secretion, and endothelial membrane re-organization to facilitate immune cell extravasation. Indeed, targeting intracellular trafficking pathways has been proposed for anti-inflammatory drug development [39]. Similarly, remodeling of the actin cytoskeleton is essential for many cellular processes involved in innate immune responses, including phagocytosis and chemotaxis of macrophages (and other immune cells) [40], and gene expression [41]. A recent report demonstrates that actin polymerization plays a critical role in limiting *Salmonella* replication within macrophages [42]. Not surprisingly, intracellular pathogens employ mechanisms to subvert the host cell cytoskeleton and promote their own survival (reviewed in [43]), such as the effector proteins encoded by *Yersinia* spp. which target the actin cytoskeleton to disrupt the phagocytic machinery of infected cell [44]. As an intracellular pathogen, *F. tularensis* utilizes both intracellular trafficking and remodeling of actin cytoskeleton to gain entry into cells. It enters macrophages via a novel process of engulfment by triggering formation of pseudopod loops utilizing complement and complement receptors [45]. It then modulates the biogenesis of the phagosome, preventing its acidification and fusion with lysosomes, to facilitate its release into the cytosol [34, 46]. It is possible that once released into the cytosol, *F. tularensis* targets intracellular trafficking and/or actin cytoskeleton to attenuate host responses which are detrimental to its survival. While it is known that *F. tularensis* infection blocks expression of an activation marker in dendritic cells [3], no study has yet investigated the effects on dendritic cell/macrophage functions including phagocytosis and antigen presentation.

That the expression of many *E.coli* LPS-responsive genes remains unaffected by *F. tularensis* infection suggests that the target is not the TLR4 receptor but rather downstream in the signaling pathway. The levels of TLR4 mRNA did not increase significantly in response to any of the *Francisella* strains relative to control animals. Exposure of FT SchuS4-infected animals to *E. coli* LPS resulted in increased expression of many MyD88-dependent genes, suggesting that this adaptor protein is not directly targeted by the bacteria. MyD88 mRNA levels increased approximately 2-fold in response to FT SchuS4 exposure alone, further increasing to 6.5-fold following *E. coli* LPS exposure. It is also unlikely that TRIF is a direct target given the relatively few genes that are strictly dependent on this adaptor protein and the extensive number of genes suppressed by *F. tularensis*. Like TLR4, TRIF mRNA levels did not increase in response to any of the *Francisella* strains. While it is possible that *F. tularensis* is antagonizing the activation of an alternative receptor/signaling pathway that is binding *E. coli* LPS, TLR4 is the central component of the LPS sensor (reviewed in [47]). Absence of TLR4 abolishes response to LPS, although cross-talk between TLR4 and TLR2 can augment this response [48]. More recently, it was shown that intracellular LPS can trigger, via an unknown mechanism, caspase-11 activation by the non-canonical inflammasome, although this requires prior stimulation with either LPS, poly (I:C), or interferons [49, 50]. Caspase-11 induces pyroptosis, a form of programmed cell death that defends against intracellular pathogens. *F. novicida* evades caspase-11 activation presumably through its unique tetra-acylated lipid A [49], but it is not known if a similar phenomenon occurs with virulent *F. tularensis* strains nor is it known if this pathway is actively inhibited. Targeting of signaling triggered by endogenous TLR4 ligands (such as HMGB1, heparan sulfate, heat shock proteins) is also a possibility, although there is uncertainty to whether these are true ligands [51].

The lack of enrichment of binding sites for the main TF activated by TLR4 signaling, including NFKB, SP1, Rel/RelA, in LPS-responsive genes suppressed by FT SchuS4 was surprising and suggests that the bacteria is targeting downstream of the immediate TLR4 signaling cascade. Overrepresentation of the binding sites for the TFs Mef2a, IRF1, CEBPA, FoxO3, HoxA5, and TBP in *E. coli* LPS-responsive genes that were inhibited in the presence of *F. tularensis* (either completely or partially) suggests their activity is modulated. While the expression levels of Mef2a and TBP were reduced in the presence of *F. tularensis* (data not shown), the others showed similar expression levels to *E. coli* LPS-treated control animals, suggesting that their activity is being modulated at the post-transcriptional level similar to what has been demonstrated for IRF1/IRF8 [10] . The expression levels of other putative transcription factors were also decreased in the presence of infection (data not shown), including many not previously linked to inflammation. Investigating the DNA binding capacity of these TF in the presence and absence of *F. tularensis* infection will more clearly define whether their activity is modulated by the bacteria and may also identify novel regulators of inflammation.

While inhibition of the pulmonary transcriptional response to *E. coli* LPS was observed at the level of whole lung, it is possible that *F. tularensis* differentially targets immune pathways in specific cell types. The majority of studies investigating the modulation of innate immune responses by *F. tularensis* have focused on macrophages and dendritic cells, the predominant infected cell type during acute infection [52]. However, the bacterium is capable of infecting multiple cell types in the lung following pulmonary exposure, including monocytes, neutrophils, alveolar type II epithelial cells [52] and endothelial cells [53] all of which express TLR4 and related adaptor proteins to varying levels [25, 54, 55]. Cell-type specific regulation of LPS-induced TLR4 signaling in the lungs is modified by microenvironmental factors including pulmonary surfactants and spatiotemporal localization of TLR4 receptor, co-receptors and adaptors (reviewed in [25]). It has been suggested that this differential regulation is important in coordinating responses to inhaled pathogens [56]. *F. tularensis* could target any of these regulatory mechanisms that fine-tune LPS signaling and may do so in a cell type-specific manner. For cell types that are the primary target for *F. tularensis* replication, including macrophages, antagonism of immune responses may be mediated primarily by factors present during bacterial replication, as suggested by the data in the current study. For cell types that are not the primary target but play a key role in activation of innate immune responses, such as lung epithelial and endothelial cells, the bacteria may utilize a secreted factor to suppress these critical responses as has been proposed [6]. Because of the likelihood of cell-type specific TLR4-mediated gene expression patterns, further studies are required to help define immune pathways that are targeted by *F. tularensis* in different cell types and determine whether these pathways are targeted by a secreted bacterial factor or factors present during intracellular bacterial replication.

## Conclusions

The present study has demonstrated that *Francisella* is capable of significantly modulating the transcriptional response to an endogenous TLR4 agonist. Thus the lack of inflammatory gene expression observed during acute infection is at least partially attributed to active suppression of this important pathway. This modulation was restricted to the highly virulent *F. tularensis* FT SchuS4 strain, highlighting the importance of using this strain when studying the interaction of virulent *Francisella* and innate immune responses. Analysis of genes inhibited by *Francisella* suggests perturbation of cellular processes including intracellular trafficking and actin cytoskeleton may broadly impact an effective innate immune response.

## Additional files

Additional file 1: Table S1. LPS-responsive genes not suppressed by FT SchuS4. (XLSX 21 kb) Additional file 2: Table S2. LPS-responsive genes partially suppressed by FT SchuS4. (XLSX 20 kb) Additional file 3: Table S3. LPS-responsive genes suppressed by FT SchuS4. (XLSX 26 kb) Additional file 4: Figure S1. A. Individual contribution of MyD88 and TRIF adaptor proteins to the pulmonary transcriptional response to aerosolized *E. coli* LPS. Wild-type, MyD88^−^/^−^, and TRIF^−^/^−^ C57BL/6 animals were exposed to aerosolized *E. coli* LPS and lung tissue harvested 2.5 h later for transcriptional profiling. Each column represents gene expression data from an individual experiment comparing RNA from lung tissue from an individual treated animal to pooled RNA isolated from untreated parental lines (*n* = 4). Sequences shown in red represent increased expression, sequences shown in green represent decreased expression and sequences in black indicate no change in expression in treated relative to control animals. Heatmap represents those sequences that are 3-fold induced (*p* value < 0.01) B. Expression profiles of LPS-responsive sequences modulated by FT SchuS4 in lung tissue from wild-type, MyD88^−^/^−^, and TRIF^−^/^−^ animals exposed to *E. coli* LPS. Each column represents gene expression data from an individual experiment comparing RNA from lung tissue from a treated animal and pooled RNA isolated from untreated parental line (*n* = 4). Sequences shown in red were increased, sequences shown in green were decreased and sequences in black indicate no change in expression in treated relative to parental control animals. (DOC 21 kb)

## Abbreviations

TLR4: Toll-like receptor 4
LPS: Lipopolysaccharide
TRIF: TIR-domain-containing adaptor-inducing interferon-β
MyD88: Myeloid differentiation primary response gene 88
TF: Transcription factor
